# Supplementary material for: Hybrid Models and Biological Model Reduction with PyDSTool
Source: PLoS Comput Biol. 2012 Aug 9;8(8):e1002628. doi: 10.1371/journal.pcbi.1002628 (PMC3415397; doi:10.1371/journal.pcbi.1002628)
Supplement: Text S4 — Complete source code for the PyDSTool package (version 0.88.120504). Includes API documentation and help files linking to web pages. This file is identical to the current public release on Sourceforge.net. (ZIP) [file pcbi.1002628.s004.zip › PyDSTool/html/PyDSTool.Toolbox-module.html]

xml version="1.0" encoding="ascii"?


PyDSTool.Toolbox


| Home | Trees | Indices | Help | PyDSTool |
| --- | --- | --- | --- | --- |

Package PyDSTool ::
Package Toolbox

[hide private]

[frames] | no frames]

# Package Toolbox

source code

Toolbox utilities for applications.

Robert Clewley, October 2005


|  |  |
| --- | --- |
| Submodules | [hide private] |

- **PyDSTool.Toolbox.ActivationFuncs**
- **PyDSTool.Toolbox.DSSRT\_tools**: *DSSRT interface tools.*
- **PyDSTool.Toolbox.FR**
- **PyDSTool.Toolbox.FSM**: *Finite State Machines.*
- **PyDSTool.Toolbox.InputProfile**
- **PyDSTool.Toolbox.ModelEst**: *Model estimation classes for ODEs.*
- **PyDSTool.Toolbox.ModelHelper**: *A set of functions to help build ODE models with complete sets of
  standard events, etc.*
- **PyDSTool.Toolbox.NineML**: *PySCes interface code for systems biology modeling and SBML model
  markup.*
- **PyDSTool.Toolbox.PRCtools**: *Toolbox for phase response curves measured by finite perturbations*
- **PyDSTool.Toolbox.ParamEst**: *Parameter estimation classes for ODEs.*
- **PyDSTool.Toolbox.PySCes\_SBML**: *PySCes interface code for systems biology modeling and SBML model
  markup.*
- **PyDSTool.Toolbox.adjointPRC**
- **PyDSTool.Toolbox.data\_analysis**: *Data analysis utilities*
- **PyDSTool.Toolbox.dataanalysis**: *Data analysis utilities*
- **PyDSTool.Toolbox.dssrt**: *Implementation of dominant scale analysis techniques for python.*
- **PyDSTool.Toolbox.event\_driven\_simulator**
- **PyDSTool.Toolbox.fracdim**: *Fractal dimension estimates for analysis of datasets having rank 2
  or 3.*
- **PyDSTool.Toolbox.makeSloppyModel**
- **PyDSTool.Toolbox.mechmatlib**
- **PyDSTool.Toolbox.model\_primitives**: *Library of common model equation primitives.*
- **PyDSTool.Toolbox.neuralcomp**: *An example set of basic compartmental model ModelSpec classes
  for use in computational neuroscience modeling.*
- **PyDSTool.Toolbox.neuro\_data**
- **PyDSTool.Toolbox.optimizers**: *Optimization module*
  - **PyDSTool.Toolbox.optimizers.criterion**: *Module containing every criteria for converge test*
    - **PyDSTool.Toolbox.optimizers.criterion.composite\_criteria**: *Composite criteria allow to use several criteria together, with
      and/or composition*
    - **PyDSTool.Toolbox.optimizers.criterion.criteria**: *A list of standard convergence criteria based on the number of
      iterations, the last values taken by the cost function and the
      associated points*
    - **PyDSTool.Toolbox.optimizers.criterion.facilities**: *Proposes a way to create a composite criterion*
    - **PyDSTool.Toolbox.optimizers.criterion.information\_criteria**
  - **PyDSTool.Toolbox.optimizers.defaults**: *Defines the defaults parameters for the generic optimizer framework*
  - **PyDSTool.Toolbox.optimizers.helpers**: *Helper functions*
    - **PyDSTool.Toolbox.optimizers.helpers.finite\_difference**
    - **PyDSTool.Toolbox.optimizers.helpers.levenberg\_marquardt**
    - **PyDSTool.Toolbox.optimizers.helpers.quadratic**
  - **PyDSTool.Toolbox.optimizers.line\_search**: *Module containing the line searchers*
    - **PyDSTool.Toolbox.optimizers.line\_search.adaptive\_last\_step\_modifier**: *Line search decorator that overrides the default alpha\_step value
      with a factor times the last alpha\_step, the factor being a
      function of the current direction and the last direction*
    - **PyDSTool.Toolbox.optimizers.line\_search.backtracking\_search**
    - **PyDSTool.Toolbox.optimizers.line\_search.barzilai\_borwein\_non\_monotone\_search**
    - **PyDSTool.Toolbox.optimizers.line\_search.barzilai\_borwein\_search**
    - **PyDSTool.Toolbox.optimizers.line\_search.cubic\_interpolation**: *Line Search with the cubic interpolation method with the
      computation of the gradient of the function*
    - **PyDSTool.Toolbox.optimizers.line\_search.damped\_line\_search**: *A damped line search*
    - **PyDSTool.Toolbox.optimizers.line\_search.fibonacci\_section**: *Line Search with the Fibonacci section method*
    - **PyDSTool.Toolbox.optimizers.line\_search.fixed\_last\_step\_modifier**: *Line search decorator that overrides the default alpha\_step value
      with a factor times the last alpha\_step*
    - **PyDSTool.Toolbox.optimizers.line\_search.golden\_section**: *Line Search with the golden section method*
    - **PyDSTool.Toolbox.optimizers.line\_search.goldstein\_rule**
    - **PyDSTool.Toolbox.optimizers.line\_search.hyperbolic\_line\_search**: *An hyperbolic line search, in fact no searches at all*
    - **PyDSTool.Toolbox.optimizers.line\_search.quadratic\_interpolation**: *Line Search with the quadratic interpolation method with the
      computation of the gradient of the function*
    - **PyDSTool.Toolbox.optimizers.line\_search.scaled\_line\_search**
    - **PyDSTool.Toolbox.optimizers.line\_search.simple\_line\_search**: *A simple line search, in fact no searches at all*
    - **PyDSTool.Toolbox.optimizers.line\_search.strong\_wolfe\_powell\_rule**
    - **PyDSTool.Toolbox.optimizers.line\_search.wolfe\_powell\_rule**
  - **PyDSTool.Toolbox.optimizers.optimizer**: *Module containing the core optimizers*
    - **PyDSTool.Toolbox.optimizers.optimizer.optimizer**: *The core optimizer from which every other optimizer is derived*
    - **PyDSTool.Toolbox.optimizers.optimizer.standard\_optimizer**: *A standard optimizer*
    - **PyDSTool.Toolbox.optimizers.optimizer.standard\_optimizer\_modifying**: *A standard optimizer with a special object that modifies the
      resulting set of parameters*
  - **PyDSTool.Toolbox.optimizers.step**: *Module containing every step use to lower a cost function*
    - **PyDSTool.Toolbox.optimizers.step.conjugate\_gradient\_step**: *Computes the conjugate gradient steps for a specific function at a
      specific point*
    - **PyDSTool.Toolbox.optimizers.step.goldfeld\_step**: *Computes Goldfeld step for a specific function at a specific point*
    - **PyDSTool.Toolbox.optimizers.step.goldstein\_price\_step**: *Computes Goldstein-Price step for a specific function at a specific
      point*
    - **PyDSTool.Toolbox.optimizers.step.gradient\_step**: *Computes a gradient step for a specific function at a specific
      point*
    - **PyDSTool.Toolbox.optimizers.step.local\_brute\_force\_1dstep**
    - **PyDSTool.Toolbox.optimizers.step.marquardt\_step**: *Computes a Marquardt step for a specific function at a specific
      point*
    - **PyDSTool.Toolbox.optimizers.step.newton\_step**: *Computes a Newton step for a specific function at a specific point*
    - **PyDSTool.Toolbox.optimizers.step.partial\_step**: *Computes a partial step for a specific function at a specific
      point, acting like a decorator for other steps*
    - **PyDSTool.Toolbox.optimizers.step.quasi\_newton\_step**: *Computes a quasi-Newton step for a specific function at a specific
      point*
    - **PyDSTool.Toolbox.optimizers.step.restart\_conjugate\_gradient**: *Restarts a conjugate gradient search by deleting the step key in
      the state dictionary*
  - **PyDSTool.Toolbox.optimizers.tests**
    - **PyDSTool.Toolbox.optimizers.tests.test\_powell**: *Class defining the Powell function*
    - **PyDSTool.Toolbox.optimizers.tests.test\_quadratic**: *Class defining a quadratic function*
    - **PyDSTool.Toolbox.optimizers.tests.test\_rosenbrock**: *Class defining the Rosenbrock function*
- **PyDSTool.Toolbox.phaseplane**: *Phase plane utilities.*
- **PyDSTool.Toolbox.prep\_boxplot**
- **PyDSTool.Toolbox.synthetic\_data**: *Helper functions for creating synthetic data*
- **PyDSTool.Toolbox.syntheticdata**: *Helper functions for creating synthetic data*
- **PyDSTool.Toolbox.test\_protocols**

  


| Home | Trees | Indices | Help | PyDSTool |
| --- | --- | --- | --- | --- |

|  |  |
| --- | --- |
| Generated by Epydoc 3.0.1 on Fri May 4 15:24:03 2012 | http://epydoc.sourceforge.net |
